# Mapping variation in intervention design: a systematic review to develop a program theory for patient navigator programs

**DOI:** 10.1186/s13643-018-0920-5

**Published:** 2019-01-08

**Authors:** Laura Desveaux, Kerry McBrien, Lianne Barnieh, Noah M. Ivers

**Affiliations:** 1 Women’s College Research Institute and Women’s College Hospital Institute for Health Systems Solutions and Virtual Care, Women’s College Hospital, 76 Grenville Ave Toronto, Toronto, Ontario M5S 1B2 Canada; 2 Institute for Health Policy, Management, and Evaluation, University of Toronto, 155 College St, Toronto, Ontario Canada; 3 Department of Family Medicine, University of Calgary, G012, Health Sciences Centre, 3330 Hospital Drive NW, Calgary, Alberta T2N 4 N1 Canada; 4 Department of Community Health Sciences, University of Calgary, TRW Building, 3280 Hospital Drive NW, Calgary, Alberta T2N 4Z6 Canada; 5 Department of Medicine, University of Calgary, 1403 29th Street NW, Calgary, Alberta T2N 2 T9 Canada; 6 Department of Family and Community Medicine, Women’s College Hospital and University of Toronto, 76 Grenville Ave, Toronto, Ontario Canada

**Keywords:** Behaviour Change Wheel, Intervention design, Patient navigator, Chronic disease

## Abstract

**Background:**

There is a great deal of variation in the design and delivery of patient navigator (PN) programs, making it difficult to design or adopt these interventions in new contexts. We (1) systematically reviewed the literature to generate a preliminary program theory to describe how patient navigator interventions are designed and delivered; and (2) describe how the resulting program theory was applied in context to inform a prototype for a patient navigator program.

**Methods:**

The current study includes a secondary review of a larger systematic review. We reviewed studies included in the primary review to identify those that designed and evaluated programs to assist patients in accessing and/or adhering to care. We conducted a content analysis of included publications to describe the barriers targeted by PN interventions and the navigator activities addressing those barriers. A program theory was constructed by mapping patient navigator activities to corresponding constructs within the capability-opportunity-motivation model of behavior change (COM-B) model of behavior change. The program theory was then presented to individuals with chronic disease, healthcare providers, and system stakeholders, and refined iteratively based on feedback.

**Results:**

Twenty one publications describing 19 patient navigator interventions were included. A total of 17 unique patient navigator activities were reported. The most common included providing education, facilitating referrals, providing social and emotional support, and supporting self-management. The majority of navigator activities targeted barriers to physical opportunity, including facilitating insurance claims, assistance with scheduling, and providing transportation. Across all interventions, navigator activities were designed to target a total of 20 patient barriers. Among interventions reporting positive effects, over two thirds targeted knowledge barriers, problems with scheduling, proactive re-scheduling following a missed appointment, and insurance. The final program design included a total of 13 navigator activities—10 informed by the original program theory and 3 unique activities informed by stakeholders.

**Conclusions:**

There is considerable heterogeneity in intervention content across patient navigator interventions. Our results provide a schema from which to develop PN interventions and illustrate how an evidence-based model was used to develop a real-world PN intervention. Our findings also highlight a critical need to improve the reporting of intervention components to facilitate translation.

**Systematic review registration:**

PROSPERO CRD42013005857

**Electronic supplementary material:**

The online version of this article (10.1186/s13643-018-0920-5) contains supplementary material, which is available to authorized users.

## Background

Patient navigation programs emerged as a promising strategy to improve access to and reduce disparities in clinical cancer care, and have since been applied across a variety of chronic conditions [1]. Patient navigators (PNs) assist individuals to overcome health system or personal barriers to care and often focus on vulnerable populations [2, 3]. We recently conducted a systematic review that identified wide variation in navigator program design across a range of chronic conditions, including the specific barriers targeted, qualifications required for the navigator role, and the measured outcome [4]. Although the heterogeneity in intervention design and diversity of implementation strategies can reflect the adaptability of patient navigator interventions, this variation presents a challenge for those seeking templates to guide the design and implementation of navigator programs.

Articulating a program theory helps to mitigate these challenges by describing the specific individual components that comprise an intervention as well as its anticipated effects [5], and serves as a tool for understanding process (i.e., how an intervention should work and why). The value of program theory in the evaluation of complex interventions is well recognized, as the quality and clarity of the underlying theory largely determine the ability to effectively scale up an intervention in new contexts [6–9].

Even with a clear program theory, the practical application of evidence is challenged by the reality that the gaps between research evidence and actual policy, practice, and health system design remain wide [10]. Stakeholder feedback, including patients, providers, operational leaders, and policy-makers, is essential to achieving engagement and ensuring feasibility. Furthermore, failure to utilize methods to optimize intervention design features increases the risk that null effects will lead to erroneous conclusions that the intervention components were incorrectly selected, when in fact they were inadequately specified or operationalized (i.e., type 3 error) [11]. The inability to definitively outline successful components remains a key barrier in applying available evidence to effectively implement PN programs in practice.

The current study is a secondary analysis of a larger systematic review that evaluated the effectiveness of PN programs, compared to usual care, in patients with chronic disease [4, 12]. Heterogeneity across interventions precluded the identification of components associated with greater effects [4]. The wide degree of variation continually observed across PN interventions [3, 4, 12, 13] highlights an opportunity and a need to systematically develop an overarching program theory of a PN intervention to guide the design and adaptation of future interventions. The objectives of this secondary analysis were to (1) create a program theory based on existing literature to describe how PN interventions for individuals with chronic disease are designed and delivered; and (2) describe how the resulting program theory was applied in context to inform a prototype for a PN program. The results informed the development of an intervention to be evaluated in a pilot trial.

## Methods

### Objective 1: Development of an evidence-based program theory

We conducted a secondary analysis of a larger systematic review [4, 12] coupled with an author-survey. We reviewed all studies included in the primary systematic review [4] to identify those that shared the primary aim with a proposed navigator program currently under development for individuals with diabetes in Alberta, Canada. The Interdisciplinary Chronic Disease Collaboration (ICDC) (www.icdc.ca), in collaboration with policy-makers, is planning to develop and implement a program where navigators work alongside individuals with diabetes to *determine barriers to accessing or adhering to care* and *facilitate the patient’s ability to overcome those barriers.* As part of this pragmatic approach, and to facilitate translation of the findings, the research questions were developed in collaboration with knowledge-user partners affiliated with the ICDC.

### Inclusion and exclusion criteria

The inclusion criteria for the primary review can be found in Table 1. Studies from the primary review were included in the secondary review if (1) the program aim involved actively working with the patient to determine barriers to accessing and/or adhering to recommended treatment; (2) the PN facilitated the patient’s ability to overcome barriers; (3) the study population was specific to individuals with diabetes OR the study population involved another chronic condition but included adherence to treatment and/or healthcare utilization as outcomes. Studies were excluded if the program aim did not align with the aim of the target program (e.g., focuses solely on screening, diagnostic resolution, or a single transition in care, or provides social support or self-management education as the only intervention component); the study population included individuals with mental illness; and a full manuscript of the study was unavailable. The decision was made to exclude studies including mental illness as this population demonstrates unique psychosocial needs and was believed to require a more intensive program [14].

**Table 1:** Inclusion criteria for the primary systematic review

Population	Adult or pediatric patients, that either had or were being screened for one of the following chronic diseases (as included in the Statistics Canada Canadian Community Health Survey): asthma, arthritis, hypertension, migraine, COPD/emphysema, diabetes, heart disease, cancer, intestinal/stomach ulcers, stroke, urinary incontinence, inflammatory bowel disorder, dementia, mood disorders, anxiety disorders; with the addition of HIV/AIDS, and chronic kidney disease, which includes transplant recipients and patients on dialysis.
Intervention	Interventions where a person with or without a healthcare-related background formally engages with patients on an individual basis to determine barriers to accessing care or following recommended guidelines. The individual also provides information relevant to patients’ specific circumstances to facilitate self-management and access to care. Interventions were excluded if the individual provided clinical care.
Comparison	Usual care (patients navigate different aspects of the health system independently and access to care is not traditionally tailored to individual barriers)
Outcome	Any

#### Definition of intervention

Aligned with the aforementioned systematic review, we defined a PN as “a person with or without a health care-related background that engages with patients on an individual basis to determine barriers to accessing care or following recommended guidelines, and provides information relevant to their specific circumstances in order to increase access to components of the health care system and to enhance their chronic disease care” ([12], p. 3). The navigator role must be formalized (e.g., navigators receiving formal training), and navigators must not provide clinical advice (e.g., medication management, interpretation of test results, advising on treatment for emergent symptoms) to distinguish between PNs and clinical case managers. Interventions referring to navigators using a different title, but meeting the criteria above, were also included.

### Data extraction and author survey

We conducted content analyses of included manuscripts describing PN interventions. Data extraction forms were created according to a hypothesized set of codes created a priori (see Additional file 1). These codes described the barriers to accessing care that were targeted by each intervention and the activities performed by PNs within each intervention. This set of codes was derived from the literature and was grouped according to three main categories, including patient-focused, system level, and other-focused barriers [15, 16]. We included an “other” category to allow for unanticipated codes.

Codes describing barriers and PN activities were extracted from the manuscript using a latent coding process. To verify the coding, the extracted codes were summarized for all included studies and sent to the corresponding author to confirm accuracy. During this phase, corresponding authors of included manuscripts were asked to provide insight regarding any additional barriers or PN activities that may have not been described in the content of the manuscript but pertained to the design of the intervention. Additional activities identified by the original manuscript author could include pre-identified codes that were not present in the manuscript (e.g., the intervention included facilitating healthcare referrals but this was not reported) or additional codes not pre-specified in the author survey (e.g., arranging transportation). Authors were also asked to identify the PN activities they felt had the greatest impact (and therefore should be included in the development of future navigator interventions) and whether an underlying theory of behavior change was used to inform the development of the intervention.

### Data analysis

Data was summarized for each study and synthesized by calculating the frequency of interventions that included a particular barrier or navigator activity. In an effort to increase the utility of the results, we further refined the intervention features reported in the primary review to be more descriptive and action-oriented PN activities (e.g., “practical assistance” in the primary review versus “link patients to financial resources” in the current study). Eleven studies (58%) reported a statistically significant difference in their primary outcome compared to the control group; unfortunately, outcome heterogeneity precluded the ability to conduct a more in-depth quantitative analysis of the data. Risk of bias was evaluated as part of the primary review and was not duplicated as part of the secondary analysis (see Additional file 2). Similar to the primary review, quality varied among studies in the current review, with 12 studies lacking information on at least 2 of the 6 criteria.

### Aligning the evidence with behavior change theory

A plethora of behavior change frameworks exist; however, the majority fail to link barriers to behavior change with evidence-based intervention strategies. We utilized the Behaviour Change Wheel (BCW) as it provides a comprehensive framework for systematically linking barriers to change to evidence-based intervention strategies [17], making it ideally suited to our objective of supporting the evidence-based design of PN interventions. Furthermore, it is presented using language that is digestible for non-scientific audiences (including patients and health system stakeholders), making it well suited to integrated, real-world application. It represents the consensus of behavior theorists following a systematic review of 19 behavior frameworks, thereby achieving scientific rigor [17]. Embedded within the BCW is the capability-opportunity-motivation model of behavior change (COM-B) that is used to conceptualize the components underlying volitional behavior change and the interactions between them. Within the COM-B system, capability refers to the “individual’s psychological and physical capacity to engage in the activity concerned [and] includes having the necessary knowledge and skills” ([17], p. 4). Motivation is defined as “all those brain processes that energize and direct behaviour, not just goals and conscious decision-making, [and] includes habitual processes, emotional responding, as well as analytical decision-making” [17].^(p.4)^ Opportunity includes “all the factors that lie outside the individual that make the behaviour possible or prompt it” ([17], p. 4).

Each targeted patient barrier was extracted and categorized according to its corresponding construct on the BCW [17], which was determined by identifying the context-dependent condition underlying the barrier. For example, poor health literacy results from a lack of psychological capability, while transportation barriers result from a lack of physical opportunity. Targeted behaviors were coded by one member of the research team (LD) and independently verified by a second member (NI).

### The role of program theory

Many challenges in achieving improvement are attributed to the failure to utilize formal and informal theory when designing interventions [18]. Understanding how and why interventions achieve an effect (or lack thereof) depends on a clearly articulated program theory that describes all the components of an intervention, and the mechanisms by which they intend to effect the desired change [9, 19]. The development and application of a program theory “enables the maximum exploitation of learning and accumulation of knowledge, and promotes the transfer of learning from one project, one context, one challenge, to the next” [9]. We utilized the COM-B as a theory-based blueprint underlying potential mechanisms of action. PN activities (intervention functions) were mapped to the COM-B to highlight the hypothesized construct targeted by each activity [17] in order to create an actionable program theory. For example, providing education targets psychological capability through the provision of knowledge; helping patients with goal setting targets reflective motivation by facilitating planning; and connecting patients with financial resources targets physical opportunity by facilitating access to resources. Linking intervention activities and targeted constructs in this way allows future implementors to understand the explicit target of their actions (i.e., to improve motivation), while future evaluations can assess the extent to which the intervention successfully influenced these targets (i.e., did patient capability improve?).

### Objective 2: Applying the program theory in context

Input was sought from a range of stakeholders and end-users according to the principles of user-centered design. Informal feedback was sought from system stakeholders engaged in the broader initiative to develop and implement a local PN program in Alberta, Canada. This included individuals involved in primary care delivery, policy-makers, clinicians, and patients, as well as individuals responsible for prioritizing areas for improvement in healthcare system and helping with knowledge translation and practical implementation. We then conducted four semi-structured focus groups with relevant end-user groups, including individuals with chronic disease and healthcare providers. The objective of the patient focus group was to explore the potential role of a PN in addressing barriers to care from the perspective of patients. The remaining focus groups were conducted with physicians (one focus group) and members of the multidisciplinary health team (two focus groups) who were involved in the provision of care. The objective of these focus groups was to draw on the patient experience and group expertise in order to transform the program theory into a navigator program prototype that could be implemented in practice.

### Recruitment

The ICDC partnered with Mosaic Primary Care Network (MPCN) to refine and implement the PN intervention. MPCN is a network of approximately 300 physicians working across approximately 90 practices in a defined geographic area of a large Canadian city (Calgary, AB). MPCN practice facilitators invited physician participants by phone, while multidisciplinary team members (all employees of MPCN) were invited to participate during regularly scheduled meeting times. Potential participants expressed interest to the practice facilitator who then arranged the focus groups. MPCN practice facilitators recruited patients through their family physician. Recruitment was led by MPCN (and not the research team) as part of their intervention development process, resulting in intentionally broad inclusion criteria requiring participants to be part of the MPCN. Focus group participants agreed to have sessions audio-recorded for the purpose of understanding how the program theory was utilized and modified in a real-world context.

### Data collection and analysis

A trained facilitator led the focus groups using a semi-structured discussion guide to facilitate review of the program theory and explore general thoughts around the intervention (Additional file 3). A translator was present at the patient focus group and provided direct translation when needed. Focus groups were audio-recorded and transcribed verbatim. Two members of the research team conducted an independent content analysis to identify themes relating to the development and operationalization of the PN intervention in the local context. Themes were discussed with the broader research team and reported according to participant group to retain context and reflect the applied nature of the study. Data that was not related to the primary objective of the focus group (obtaining feedback on the role of a PN in addressing barriers to care) will be reported separately.

## Results

### Objective 1: Evidence-based program theory

**Fig. 1:**
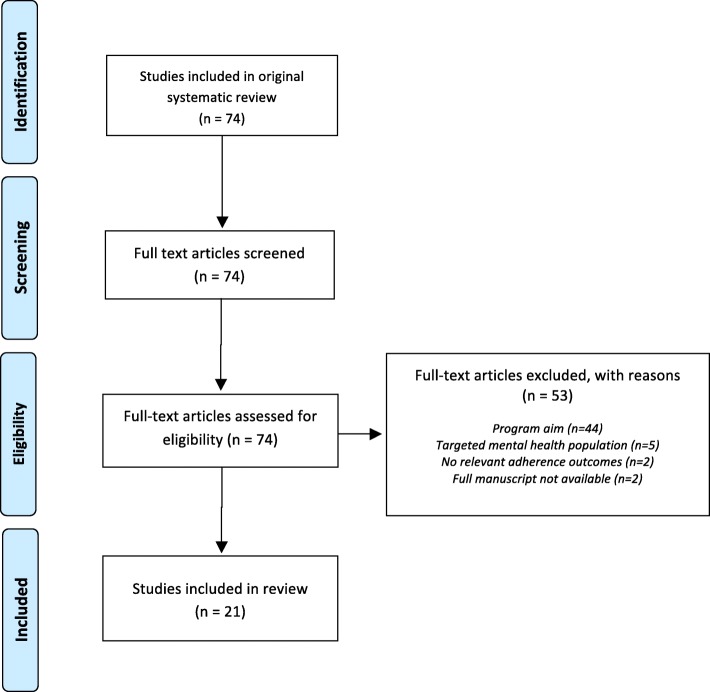
Study flow diagram

Of the 74 publications included in the primary review, 21 publications met the inclusion criteria for the current review (Fig. 1). Reasons for exclusion included program aim (*n* = 44), mental health as a target condition (*n* = 5), no relevant adherence outcomes (*n* = 2), and no full manuscript available (*n* = 2). Seven publications included individuals with diabetes as the target population [20–28], while the remaining publications targeted cancer [29–31], HIV [32–34], kidney failure [35], and a range of chronic health conditions (refer to Additional file 4 for individual study details) [36]. As three publications evaluated the same intervention with different outcomes [20–22], the unit of analysis became the intervention rather than the article, resulting in a total of 19 PN interventions. In addition to PNs, individuals delivering the intervention were also referred to as case managers [29, 32–34], care ambassadors [25, 27], community health workers [23, 24, 26, 28], public health nurses [36], and health coaches [20–22]. Of the 12 publications including adherence as an outcome, 9 reported significant improvements [22, 25, 27–29, 32, 34–36], while seven of 11 reported significant improvements in disease-specific patient outcomes [20, 21, 23–25, 27, 36]. Studies were more likely to report positive results for process measures, and less so for surrogate markers or patient-level outcomes, although the latter were more common as outcomes. No studies found a negative impact from the PN intervention.

### Patient navigator activities

A total of 17 action-oriented PN activities were reported across the 19 interventions, compared with the 12 intervention features reported in the primary review. Twelve of the reported PN activities were included in at least half of the interventions (Tables 2 and 3). Of the 148 activities reported across the 19 interventions, 110 were extracted during the content analysis while 38 (26%) were reported through communication with the authors directly. Providing education, facilitating healthcare referrals, providing social and emotional support, and supporting self-management were the most common responsibilities of PNs, with education featured in over two-thirds of interventions with positive effects. Helping patients with goal setting and linking them to billing/insurance personnel or financial resources were most likely to be omitted from the manuscript. Authors reported that providing education, social and emotional support, and helping patients with goal setting had the greatest perceived impact on participants and should be included in future interventions. Context-dependent activities, including arranging transportation and acting as an interpreter, were included in two studies, while liaising with the patient’s employer was included in one study directed at women enrolled in a welfare-to-work program.

**Table 2:** Patient navigator activities across included studies for individuals with diabetes

	Thom 2014/2015, Willard-Grace 2015	Prezio 2013	Spencer 2011	Svoren 2003	Gary 2003	Laffel 1998	Corkery 1997^a^
Provide education (written or verbal)	^b^	^b^	^c^	^b^	^b^		
Schedule healthcare appointments	^b^	^b^		^b^	^b^	^b^	^b^
Attend patient appointments	^b^		^c^				^b^
Facilitate healthcare referrals	^b^	^b^	^b^	^b^			
Improve communication with HCP	^b^		^b^	^c^			
Provide information to HCP	^b^				^b^		
Act as an interpreter	^c^						^b^
Support self-management	^b^	^b^	^b^		^b^		^b^
Provide social and emotional support	^b^		^b^		^b^		
Help patients with goal setting	^b^		^b^	^c^	^c^		
Link patients to social resources	^b^		^c^		^c^		
Link patients to billing/insurance personnel				^b^		^b^	
Link patients to financial resources					^c^		
Liaise with employer to ensure health needs are met							
Monitor attendance and follow-up after missed appointments	^c^			^b^		^b^	^b^
Other							

*HCP* healthcare provider

^a^Data from Corkery et al. extracted via content analysis only as attempts to contact the author were unsuccessful

^b^Information extracted from content analysis of published manuscript

^c^Additional information provided via direct correspondence with author

**Table 3:** Patient navigator activities across included studies for individuals with other conditions

	Navaneethan 2017	Metsch 2016	Giordano 2016	Bassett 2016^a^	Percac-Lima 2015	Metsch 2015	Fiscella 2012	Sullivan 2012	Kneipp 2011	Ell 2009	Wohl 2006	Gardner 2005
Provide education (written or verbal)	^b^	^b^	^b^	^b^		^b^	^b^	^b^	^b^	^b^	^b^	^c^
Schedule healthcare appointments	^b^	^b^	^b^			^b^	^b^		^b^			
Attend patient appointments	^b^	^b^				^c^	^b^		^b^			^c^
Facilitate healthcare referrals	^b^	^b^				^b^	^c^	^b^	^c^		^b^	^b^
Improve communication with HCP							^b^	^b^	^c^		^b^	
Provide information to HCP	^b^	^b^			^b^		^b^		^c^		^b^	^c^
Act as an interpreter												
Support self-management	^b^	^b^	^b^	^b^		^b^			^c^	^b^	^b^	^b^
Provide social and emotional support	^b^	^b^	^b^	^b^		^c^	^c^	^b^	^b^	^c^	^b^	^b^
Help patients with goal setting		^b^	^b^			^c^		^c^	^b^		^b^	^c^
Link patients to social resources	^b^	^b^			^c^	^b^	^b^	^c^	^b^		^b^	
Link patients to billing/insurance personnel	^b^	^b^			^c^	^b^	^c^	^c^	^c^		^b^	^c^
Link patients to financial resources	^b^				^c^	^c^	^b^	^c^	^c^		^b^	
Liaise with employer to ensure health needs are met									^b^			
Monitor attendance and follow-up after missed appointments							^c^	^b^	^c^			
Other					^b^ Appointment reminders	^b^ Arrange transportation		^b^ Arrange transportation				

*HCP* healthcare provider

^a^Data from Bassett et al. extracted via content analysis only as attempts to contact the author were unsuccessful

^b^Information extracted from content analysis of published manuscript

^c^Additional information provided via direct correspondence with author

#### Patient barriers targeted by navigator interventions

Twenty barriers were reported across all included interventions, with an average of 8 barriers addressed by each intervention (Table 4) [29–36]. A wide variation was observed, with a minimum of three and a maximum of 17 barriers targeted per intervention overall. Patient knowledge was explicitly targeted as a barrier in all but one intervention [27]. Insurance, social support, and problems with scheduling were targeted in over two thirds of studies. Among interventions reporting positive effects, over two thirds targeted knowledge, problems with scheduling, facilitating a proactive system, and insurance. Housing, financial barriers, employment demands, attitudes toward healthcare providers, and comorbidities were targeted in less than one-third of interventions reporting a positive effect on outcomes.

**Table 4:** Barriers targeted by patient navigator interventions

	Diabetes studies							Other conditions												Interventions targeting barrier (%)
	Thom 2014 Thom 2015 Willard-Grace 2015	Prezio 2013	Spencer 2011	Svoren 2003	Gary 2003	Laffel 1998	Corkery 1997^a^	Navaneethan 2017	Metsch 2016	Giordano 2016	Bassett 2016	Percac-Lima 2015	Metsch 2015	Fiscella 2012	Sullivan 2012	Kneipp 2011	Ell 2009	Wohl 2006	Gardner 2005	
Capability																				
Psychological																				
Literacy	♦	♦	♦					♦	♦	♦			♦	♦				♦		47
Language	♦	♦	♦				♦				♦	♦		♦			♦	♦	♦	53
Knowledge	♦	♦	♦	♦	♦		♦	♦	♦	♦	♦	♦	♦	♦	♦	♦	♦	♦	♦	95
Medical/mental health comorbidities	♦				♦			♦				♦				♦		♦		32
Physical																				
Communication with HCPs	♦		♦		♦			♦	♦				♦	♦	♦	♦		♦		53
Disability								♦						♦		♦				16
Opportunity																				
Physical																				
Problems with scheduling	♦	♦		♦	♦	♦	♦	♦			♦	♦	♦	♦		♦				63
System proactive	♦		♦	♦		♦	♦	♦	♦		♦		♦	♦	♦	♦				63
Insurance		♦	♦	♦	♦	♦		♦	♦				♦	♦	♦	♦		♦	♦	68
Financial	♦				♦			♦						♦		♦		♦		32
Employment demands					♦							♦		♦		♦		♦		26
Housing	♦				♦									♦				♦		21
Transportation	♦				♦			♦	♦				♦	♦	♦			♦	♦	47
Location of facility								♦						♦						11
Child care					♦				♦									♦		26
Social																				
Social support	♦		♦		♦			♦	♦	♦	♦		♦	♦	♦	♦	♦	♦	♦	74
Motivation																				
Automatic																				
Fear			♦		♦								♦	♦	♦	♦			♦	37
Reflective																				
Care is not a priority	♦								♦	♦		♦	♦		♦	♦			♦	42
Attitudes toward HCPs			♦		♦									♦		♦			♦	26
Perceptions about treatment	♦		♦		♦							♦		♦	♦	♦			♦	42

*HCP* healthcare provider

^a^Data extracted from manuscript, unable to confirm with author

♦The barrier was targeted in the study

#### Program theory development

**Fig. 2:**
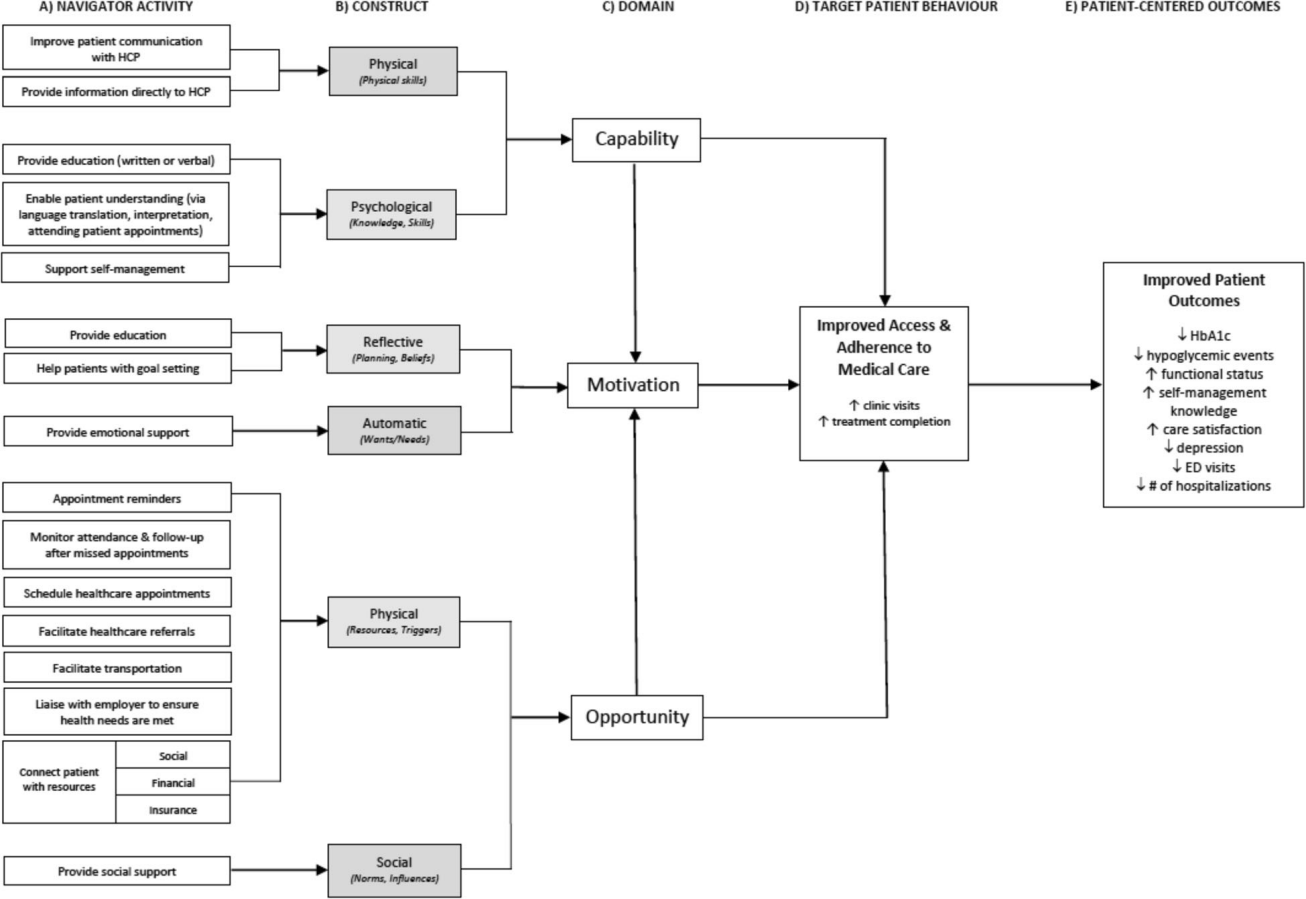
Composite program theory underlying patient navigator interventions (*n* = 19). Potential patient navigator activities (**a**) are linked to the corresponding behavior construct that they target (**b**). These constructs are components of more generalized sources (**c**) that influence individual behavior. These sources directly influence the overall health behavior targeted by patient navigator interventions (**d**), which has a direct impact on patient-centered outcomes (**e**). Note: All outcomes listed were shown to be significantly impacted by patient navigator interventions. ED = emergency department; HCP = healthcare professional

A total of 17 PN activities were mapped to the constructs underlying behavior change as described in the COM-B model [17]. All six sub-constructs of the COM-B behavioral model were targeted by at least one navigator activity. The majority of activities were intended to improve the patient’s physical opportunity to adhere to recommended treatment (i.e., by facilitating referrals, scheduling appointments, and monitoring attendance). The provision of education, emotional support, and assistance with goal setting were intended to target individual motivation by addressing the patient’s beliefs and needs. Several activities aimed to overcome barriers relating to individual capabilities, including knowledge and understanding, the ability to communicate effectively with healthcare providers, and the ability to self-manage their chronic condition. Through targeting these proximal constructs, the PN activities aimed to improve the patient’s access to care and adherence to recommended treatment (the target behavior). Figure 2.

### Objective 2: Applying the program theory in context

#### Feedback from system stakeholders

**Fig. 3:**
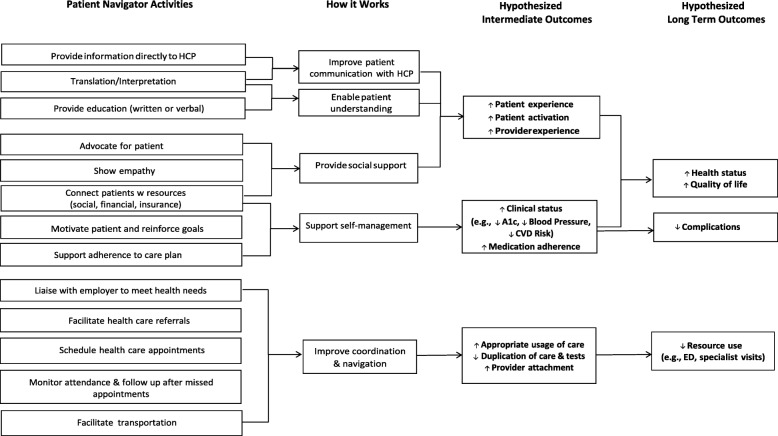
Final applied program theory of patient navigator intervention

Health system stakeholders expressed that a PN program should be implemented in primary care in order to optimize patient engagement and system integration. Stakeholders advised that evidence-based constructs should be described in terms of what they mean for the PN in practice—specifically, what they are intended to directly influence for the patient. As a result, intermediate outcomes were added to the program theory (Fig. 3). Stakeholders also advised to expand the program beyond diabetes to include individuals with a diagnosis of two or more chronic conditions in order to align with broader system goals. Program inclusion criteria were modified in response.

#### Feedback from patients

Patients described challenges navigating the different components of their local health system and expressed a strong desire for a PN to help with scheduling appointments, managing referrals, and providing appointment reminders. Many patients experienced language barriers and emphasized the importance of an interpreter who can help them understand the medical process and the rationale behind it. As this communication barrier exists across many healthcare provider relationships, the PN would ideally help the patient improve communication with all providers involved in their care. Patients expressed a need for education about the local health system and how to navigate it, as well as support with securing and/or accessing transportation options to ensure they were able to attend their appointments.

#### Feedback from physicians and multidisciplinary team members

Healthcare providers continuously expressed the need for role clarity so that both the patient and the providers involved in their care understood the PN’s scope. Overall, healthcare providers felt the PNs overarching responsibilities were to coordinate appointments and support self-management and system navigation. Their role was described as “task-oriented,” whereby they can support the patient’s care by connecting them with required resources and provide general support. Echoing the patient perspective, healthcare providers expressed a need for PNs to assist with appointment scheduling and follow-up and arranging transportation to visits. Facilitating communication would add significant value, including ensuring that all healthcare team members are kept “in the loop” for more complex patients. Translation skills were a key requirement in order to address communication needs and educate the patient on how to navigate the different features of the local health system.

## Discussion

Our results highlight the substantial heterogeneity in both intervention content and terminology used to describe PNs. Heterogeneity across interventions, even across a single condition, is likely attributable to the complex nature of navigator interventions, since multiple intervention components may be needed to achieve an impact on the desired outcome(s). Faced with this reality, health system administrators and healthcare providers may struggle to decide which components to use to address particular barriers in a given population.

Provision of education and facilitation of healthcare referrals were navigator responsibilities in the majority of interventions reporting positive effects. This aligns with previous qualitative work that found that identifying and overcoming system barriers, including the ability to integrate care within fragmented healthcare system, contribute to successful PN interventions [37, 38]. Jean-Pierre et al. [37] also found that relationship interventions (e.g., enhancing patient-navigator and patient-provider relationships) contribute to success. Yet, we found that only 55% of interventions reporting positive effects described providing social support while 45% reported improving communication with healthcare providers. Further, the four interventions that failed to demonstrate an effect reported providing social and emotional support to participants [26, 30, 31, 33]. This finding may reflect variations in the provision of social support: advising on or arranging general social support to provide encouragement and counseling directed at the behavior (e.g., asking family members to encourage the patient to continue with treatment); practical support to aid in performing the behavior (e.g., ask a friend or relative to leave the patient’s medication out for them each morning); or emotional social support relating to the performance of the behavior (e.g., asking friends, relatives, or colleagues to attend the appointment with the patient) [39].

PN interventions can effectively reduce barriers to care [40, 41]. However, few studies have systematically described the specific actions of a PN or the mechanism(s) by which navigator interventions impact these barriers or overall clinical outcomes [37]. The program theory developed in this study provides an integrated summary of navigator activities and the proposed causal processes by which these activities influence patient behavior and impact patient outcomes. In doing so, the program theory offers a standardized and systematic framework for designing and evaluating PN interventions and their content. We believe that it may also be useful to facilitate engagement with a variety of stakeholders in the course of an intervention design process.

To use the program theory effectively, users should first identify the behavioral target(s) of the proposed intervention (e.g., improved adherence to prescribed treatment). Once the target behavior is identified, users can work from right to left across the theory to identify the appropriate behavioral domains the intervention will be designed to target, and ultimately select the specific PN activities that correspond to the selected domains. The domains and their constructs comprise the COM-B system, which provides a simplified framework for understanding behavior [17]. The program theory was created with the intention that users would consider their patient population and the specific context(s) influencing their interaction with the health system and subsequently identify potential intervention components relevant to their population. Depending on the needs of those developing or evaluating the intervention, it might be supplemented by a formal (i.e., mid-level) theory relevant for the targeted behavior or context. The program theory can also support the tailoring of intervention content to ensure it is evidence-based and appropriately targeting patient behavior(s). Finally, the program theory serves to inform the selection of appropriate proximal measures to evaluate the impact of patient navigation programs in practice.

## Limitations

Understanding how to optimize components within complex interventions requires detailed reporting of those components. Unfortunately, we observed underreporting of intervention details across included studies. Although this phenomenon is not unique to PN interventions [42, 43], inadequate descriptions constrain scientific replication of study results and the subsequent introduction of effective interventions within the healthcare system. As a result, interventions may be used incorrectly or fail to be used at all [8].

In light of the systematic underreporting of intervention components, there may be details that were not captured as part of this review. This was mitigated by verifying extracted codes with authors and requesting additional information where possible. Furthermore, we may not have captured all relevant publications due to the variation in terminology used to describe PN interventions and the exclusion of non-randomized studies. We surveyed authors of included studies to validate our data extraction and address the potential implications of incomplete reporting highlighted in the primary review. Despite this, the relatively small number of publications and outcome heterogeneity precluded the ability to conduct a meta-analysis to identify key components underlying intervention effectiveness. Outcome results have been described for completeness; however, conclusions should not be drawn regarding effectiveness. Variation in how the individual features were implemented across studies further complicates the identification of key elements. Future studies should focus on not only what components were included as part of PN interventions, but also how they were operationalized in practice. Although the number of included studies reporting significant versus insignificant findings appeared balanced, we are unable to rule out publication bias.

The program theory may also be limited by the choice of behavioral theory. The COM-B was selected as the language is easily understood by non-scientific audiences, and it provides the practical advantage of linking barriers to specific intervention strategies. By using a comprehensive framework, we sacrificed the ability to obtain a more nuanced understanding of behavioral factors that more specific behavioral theories may have afforded. Finally, it is important to note that our findings may not apply to PN interventions addressing clinical issues outside the scope of this review where the targeted patient behaviors and/or associated barriers may be different.

## Conclusions

The patient navigation program theory developed through this review provides a theory-informed foundation that can facilitate both the evidence-based design and subsequent evaluation of PN interventions. The practical application of the theory helps to illustrate how contextual factors can be incorporated into program theories to increase the integration of evidence into the design of health system solutions. The results of this review highlight a critical need to improve the reporting of intervention components to allow replication and ensure transparency around how and why the intervention was actually delivered. Future evaluations should explicitly outline the activities of PNs and link these actions to evidence-based intervention functions.

## Additional files

Additional file 1: Data extraction form with author responses. (PDF 846 kb)

Additional file 2: Risk of bias assessment results from primary study. (DOCX 32 kb)

Additional file 3: Focus group question guide. (DOCX 15 kb)

Additional file 4: Description of included studies. (DOCX 17 kb)

## Abbreviations

BCW: Behaviour Change Wheel
COM-B: Capability-opportunity-motivation model of behavior change
PN: Patient navigator
