# Supplementary material for: Mapping variation in intervention design: a systematic review to develop a program theory for patient navigator programs
Source: Syst Rev. 2019 Jan 8;8:8. doi: 10.1186/s13643-018-0920-5 (PMC6323765; doi:10.1186/s13643-018-0920-5)
Supplement: Supplementary file 2 — Risk of bias assessment results from primary study. (DOCX 32 kb) [file 13643_2018_920_MOESM2_ESM.docx]

**Additional File 2: Risk of Bias of Included Studies**

| **Study** | **Random sequence generation** | **Allocation concealment** | **Blinding of outcome assessment** | **Incomplete outcome data** | **Group similarity at baseline** | **Intention-to-treat analysis** |
| --- | --- | --- | --- | --- | --- | --- |
| Corkery (1997) |  |  |  |  |  |  |
| Laffel (1998) |  |  |  |  |  |  |
| Gary  (2003) |  |  |  |  |  |  |
| Svoren (2003) |  |  |  |  |  |  |
| Spencer (2011) |  |  |  |  |  |  |
| Prezio (2013) |  |  |  |  |  |  |
| Thom (2014) |  |  |  |  |  |  |
| Gardner (2005) |  |  |  |  |  |  |
| Wohl  (2006) |  |  |  |  |  |  |
| Ell  (2009) |  |  |  |  |  |  |
| Kneipp (2011) |  |  |  |  |  |  |
| White (2011) |  |  |  |  |  |  |
| Fiscella (2012) |  |  |  |  |  |  |
| Sullivan (2012) |  |  |  |  |  |  |
| Metsch (2015) |  |  |  |  |  |  |
| Percac-Lima (2015) |  |  |  |  |  |  |
| Shaw  (2016) |  |  |  |  |  |  |
| Metsch (2016) |  |  |  |  |  |  |
| Giordano (2016) |  |  |  |  |  |  |
| Bassett (2016) |  |  |  |  |  |  |
| Navaneethan (2017) |  |  |  |  |  |  |

|  | Low risk of bias |
| --- | --- |

|  | Unclear risk of bias |
| --- | --- |

|  | High risk of bias |
| --- | --- |
