# Supplementary material for: Mapping variation in intervention design: a systematic review to develop a program theory for patient navigator programs
Source: Syst Rev. 2019 Jan 8;8:8. doi: 10.1186/s13643-018-0920-5 (PMC6323765; doi:10.1186/s13643-018-0920-5)
Supplement: Supplementary file 3 — Focus group question guide. (DOCX 15 kb) [file 13643_2018_920_MOESM3_ESM.docx]

**Additional File 3- Focus Group Questions**

1. *For patients only:* What barriers do you experience when navigating the health system?
2. Let’s review the proposed patient navigator activities. Which ones do you feel would be beneficial and why?
3. What do you think are the most important aspects?
4. What activities do you feel aren’t necessary? Why?
5. Is there anything that isn’t currently included in the model that you think should be included? Why?
6. *For healthcare providers only:* How do you envision the navigators/community health workers fitting in to existing healthcare teams?
7. Are there any additional comments or anything we have missed?
