# Supplementary material for: Mapping variation in intervention design: a systematic review to develop a program theory for patient navigator programs
Source: Syst Rev. 2019 Jan 8;8:8. doi: 10.1186/s13643-018-0920-5 (PMC6323765; doi:10.1186/s13643-018-0920-5)
Supplement: Supplementary file 4 — Description of included studies. (DOCX 17 kb) [file 13643_2018_920_MOESM4_ESM.docx]

**Additional File 1: Description of Included Studies**

| **Study** | **Population** | **Intervention Aim** | **Between-Group Comparisons** | **Theory Underlying Intervention** |
| --- | --- | --- | --- | --- |
| **Diabetes** | | | | |
| Willard-Grace 2015  Thom 2015  Thom 2014 |  | Inform, enable, and reinforce connections to patient assistance program in order to improve control of one or more chronic vascular disease risk factors not controlled at baseline. | **Adherence**  -# clinic visits (+ p<0.001)  -medication adherence (+ p<0.001)  -medication concordance (+ p=0.013)  **Patient Outcomes**  -glycemic control (HbA_1C_) (+ p=0.01)  -improved control of all conditions uncontrolled at baseline (+ p=0.05)  -physician trust (+ p=0.047) | 1. Chronic Care Model  2. Transtheoretical Model of Behaviour Change  3. Self-efficacy  4. Theory of Planned Behavior 5. Social Cognitive Theory  6. Pender's Health Promotion Model |
| Prezio 2013 | Mexican Americans  Control  n=90, mean age 46 ± 11  Intervention  n=90, mean age 48 ± 11 | Provide access to diabetes self-management education for uninsured patients. | **Patient Outcomes**  -glycemic control (HbA_1C_) (+ p=0.02)  -lipids (-)  -BP (-)  -BMI (-) | 1.Social Cognitive Theory |
| Spencer 2011 | Hispanic & African Americans  Control  n=92, mean age 55 ± 1  Intervention  n=72, mean age 50 ± 1 | Improve HbA_1C_ and diabetes self-management behaviours. | **Patient Outcomes**  -HbA_1C_ (+ p<0.01)  -lipids (-)  -BP (-)  -BMI (-)  -self-management knowledge (+ p<0.01)  -self-management action (-)  -psychological distress (-)  -self-efficacy (-) | 1.Empowerment Theory  2. Social Ecological Theory |
| Svoren 2003 | Children and Adolescents  Control  n=108, mean age 12 ± 3 years  PN Intervention  n=94, mean age 12 ± 2 years  PN+education Intervention  n=97, mean age 12 ± 2 years | Help patients and their families receive ambulatory diabetes care as prescribed by the patient’s usual diabetes health care team.  Note: PN+education group also received 8 psychoeducational modules relating to diabetes care. Modules included 2-3 pages of written information which was reviewed & discussed with the PN. | *PN+education & PN vs. Control*  **Adherence**  -# clinic visits (+ p=0.0001)  *PN+education vs. PN & Control*  **Healthcare Utilization**  -# hospitalizations (+ p=0.04)  -# of emergency room visits (+ p=0.004)  **Patient Outcomes**  -HbA_1C_ (+ p=0.01)  -number of significant hypoglycemic events (+ p=0.01) | None stated. |
| Gary 2003 | African Americans  Control  n=34, mean age 57 ± 8  Intervention  n=41, mean age 59 ± 9 | Improve diabetic control. | **Patient Outcomes**  -HbA_1C_ (-)  -lipids (-)  -BP (-)  -dietary practice (-)  -leisure time physical activity (-) | 1. Precede-Proceed |
| Laffel 1998 | Children  Control  n=82, mean age 13 ± 1  Intervention  n=89, mean age 13 ± 1 | Help patients and their families receive ambulatory diabetes care as prescribed by the patient’s usual diabetes health care team. | **Adherence**  -# clinic visits (+ p=0.0001)  **Healthcare Utilization**  -hospitalization/ED visits (+ p=0.034)  **Patient Outcomes**  - HbA_1C_ (+ p=0.039)  -hypoglycemic events (+ p=0.009) | None stated. |
| Corkery 1997 | Hispanic & African Americans  Total Sample*  mean age 53 ± 12  Control  n=34  Intervention  n=30 | Facilitate completion of a diabetes education program. | **Adherence**  -education completion (+ p=0.01)  **Patient Outcomes**  -HbA1C (-)  -knowledge (-)  -self-care behaviour (-) | None stated. |
| **Cancer** | | | | |
| Percac-Lima 2015 | Control  n=1603, no mean age provided  Intervention  n=1631, no mean age provided | Improve outpatient compliance with appointments for patients with cancer who were identified by predictive modeling as being at high risk of missing a scheduled appointment. | **Adherence**  -reduction in appointment no-shows (+ p<0.001) | None stated. |
| Fiscella 2012 | Total Sample*  mean age 57  Control  n=213  Intervention  n=225 | Optimize treatment of patients diagnosed with cancer by providing a PN to act as a guide and coach during cancer treatment. | **Adherence**  -time to completion of Rx (-)  **Patient Outcomes**  -psychological distress (-)  -care satisfaction (-)  Note: being randomized to navigation was associated with significantly greater likelihood of higher care satisfaction among participants with lower English proficiency, less than a high school education, and no health insurance | 1. Self-Determination Theory |
| Ell 2009 | Women  Control  n=239, no mean age provided  Intervention  n=248, no mean age provided | Improve treatment and follow-up access and adherence by influencing predisposing (knowledge/attitudes), reinforcing (social support/cues to action), and enabling (barrier reduction skill). | **Adherence**  -Rx adherence (-)  -medication adherence (-)  **Patient Outcomes**  -BPI (-)  -functional status (-)  -anxiety (-)  -depression (-)  -survival (-)  -HRQL (-) | 1. Health Belief Model  2. Socio-cultural Explanatory Theory |
| **HIV** | | | | |
| Bassett 2016 | Newly diagnosed HIV-infected out- patients in Durban, South Africa  Total sample  Mean age 35±10  Control  n = 923, mean age 35±10  Intervention  n = 967, mean age 35±10 | Improve linkage to HIV and tuberculosis (TB) care among newly diagnosed HIV-infected outpatients in Durban, South Africa. | **Adherence**  Completing at least 3 months on antiretroviral therapy or completing 6 months of TB treatment (-)  **Patient Outcomes**  Death (-) | 1. Andersen model of health services utilization |
| Giordano 2016 | Adults newly diagnosed with HIV infection or out of care.  Total sample  Mean age NR  Control  n = 215, mean age NR  Intervention  n = 202, mean age NR | Improve viral load and retention in outpatient HIV care of hospitalized, newly diagnosed or out-of-care HIV infected persons | **Patient Outcomes**  Viral load improvement (-)  **ART and adherence measures**  -prescribed ART (-)  -taking ART (-)  -adherence to ART (-)  -Retention in care (-)  **Healthcare utilization**  Hospitalized at least once (-)  ER visit (-) | 1. Information, motivation, and behavioral (IMB) skills health behavior model |
| Metsch 2015 | Total sample Median age 44  Control  n=302, mean age NR  Intervention  n=293, mean age NR | Increase access to and use of oral health services among low-income individuals with HIV. | **Adherence**  -# visits at 6- and 12-month follow-up (+ p<0.001 and p=0.011, respectively) | 1. Self-Efficacy/Strengths  2. Perspective Theory  3. Health Services Utilization Model |
| Metsch 2016 | Adults with HIV-1 viral loads and substance use  Total sample  Median age 44.6  Control  n = 264, mean age 44.4±10  Intervention 1 (Navigation +Incentives)  n = 271, mean age 44.7±10  Intervention 2  (Navigation only)  n = 266, mean age 44.8±10 | Achieve targeted behaviors aimed at reducing substance use, increasing engagement in HIV care, and improving HIV outcomes. | Outcomes reported at 12 months  **Adherence**  -ART pills taken in last mo (-)  **Patient Outcomes**  -Treatment Success (-)  -Viral suppression (success) (-)  -Death (-)  -Substance use (-)  -Take HIV medications, medical records, Navigation + incentives vs. usual treatment (p = 0.04), self-report (p=0.06)  -Visited HIV specialist (-)  -Substance use disorder treatment (-)  -Hospitalizations (-) | None stated. |
| Wohl 2006 | Control  n=84, mean age NR  Intervention  n=84, mean age NR | Use a brief case management intervention to improve adherence to antiretroviral therapy among persons living with HIV. | **Adherence**  -% of patients who did not miss a dose (-)  -viral load (-)  **Patient Outcomes**  -CD4 count (-)  -development of new infection (-) | 1. Transtheoretical Model of Behaviour Change |
| Gardner 2005 | Control  n=137, mean age NR  Intervention  n=157, mean age NR | Link HIV infected persons to HIV medical care, and to sustain this linkage for more than a single visit. | **Adherence**  -self-reported attendance within first 6 months (+ p=0.0005) and 12 months (+ p=0.006)  Note: ↓ viral load (+ p=0.02) *for those linked to care in either group* | 1. Self-Efficacy/Strengths  2. Perspective Theory |
| **Kidney Disease** | | | | |
| Navaneethan 2017 | Control  n=57, mean age NR  Intervention 1 (Patient navigation)  n=53, mean age NR  Intervention 2  (Enhanced Personal Health Record)  n=50, mean age NR  Intervention 3 (both)  n=69 mean age NR | Develop a navigator program for patients with CKD and an electronic health record–based enhanced personal health record to disseminate CKD stage–specific goals of care and education to improve kidney and overall heath. | **Patient outcomes**  Decline in eGFR (-)  Reached next CKD stage, started dialysis, or got transplant (-)  HbA1c, N (%) - Usual care significantly different from enhanced personal health record group (+p=0.02)  Hemoglobin - All other groups significantly different from enhanced personal health record group (+p=0.02)  Serum creatinine - All other groups significantly different from enhanced personal health record group (+p<0.001)  LDL Cholesterol (-)  Serum phosphorus (-)  Vitamin D (-)  PTH (-)  Albumin:Creatine (-)  **Healthcare Utilization**  No. of hospitalizations/ED visits (-) | 1. The Chronic Care Model |
| Sullivan 2012 | Control  n=75, mean age NR  Intervention  n=92, mean age NR | Help patients complete transplant process steps in a more efficient and equitable manner. | **Adherence**  -completion of steps (+ p<0.001) | None. |
| **Chronic Disease** | | | | |
| Kneipp 2011 | Women  Control  n=218, mean age 31 ± 9  Intervention  n=214, mean age 29 ± 7 | Increase rates of health care visits for mental health and chronic health conditions and increase the ability to navigate the Medicaid system. Improve mental and physical health and employment outcomes for women enrolled in the welfare-to-work program. | **Adherence**  -visit rate (+ p=0.007)  **Patient Outcomes**  -HRQL (-)  -depression (+)  -functional status (+)  -Medicaid knowledge (+ in both intervention and control) | 1. Transactional Model of Stress and Coping  2. Milio’s Ecological Framework |

Note: Mean age reported in years. Standard deviations reported when present in original manuscript.

*Mean age reported for total sample only.

BPI=Brief pain inventory; HCP=healthcare provider; HRQL=health-related quality of life; NR=not reported; PN=patient navigator.
