# Supplementary material for: Mapping variation in intervention design: a systematic review to develop a program theory for patient navigator programs
Source: Syst Rev. 2019 Jan 8;8:8. doi: 10.1186/s13643-018-0920-5 (PMC6323765; doi:10.1186/s13643-018-0920-5)
Supplement: Supplementary file 1 — Data extraction form with author responses. (PDF 846 kb) [file 13643_2018_920_MOESM1_ESM.pdf]

Willard-Grace et al. 2013. The effectiveness of medical assistant health coaching for low-income patients with uncontrolled diabetes, hypertension, and hyperlipidemia: protocol for a randomized controlled trial and baseline characteristics of the study population. ***BMC Family Practice.*** **14(27):1-10.**

Protocol for Thom 2014, Thom 2015, and Willard-Grace 2015.

Below please find a summary of the details we have extracted from your manuscript. Please confirm that we have reported accurate descriptions of the intervention aim and the actions taken by the health coach. In addition, please confirm whether we have accurately indicated all of the barriers targeted by the intervention, or whether additional barriers were also addressed. There are four (4) questions in total.

To test the hypothesis that in-clinic health coaching by medical assistants trained as health coaches improves control of one or more chronic vascular disease risk factors (diabetes, hypertension or hyperlipidemia) not controlled at baseline.

1. Intervention Aim: Inform, enable, and reinforce connections to patient assistance programs.

2. Health Coach Duties: (Please check the boxes below to confirm the duties we extracted from the manuscript and to identify those we may have missed)

- ☒ Provide education (written or verbal)
- ☒ Attend patient appointments
- ☒ Improve patient communication
- ☒ Act as an interpreter
- ☒ Provide social/emotional support
- ☒ Link patients to social resources
- ☐ Link patients to financial resources
- ☒ Schedule visits
- ☒ Facilitate referrals
- ☒ Provide information to healthcare provider
- ☒ Support self-management/reinforce skills
- ☒ Help with patient goal setting
- ☐ Link patients to billing/insurance personnel
- ☐ Liaise with employer to make sure health needs are met
- ☒ Monitor attendance & follow-up after missed appointments
- ☐ Other:

Of the duties listed above, please identify those which you feel had the greatest impact or should be included in the development of future health coaching interventions (list below or highlight above).

Education, social/emotional support, Support self-management/reinforce skills, Support self-management/reinforce skills

Willard-Grace et al. 2013. The effectiveness of medical assistant health coaching for low-income patients with uncontrolled diabetes, hypertension, and hyperlipidemia: protocol for a randomized controlled trial and baseline characteristics of the study population. *BMC Family Practice.* 14(27):1-10.

Protocol for Thom 2014, Thom 2015, and Willard-Grace 2015.

3. Barriers targeted by program: (Please check the boxes below to confirm that we have accurately identified the barriers targeted by the intervention and to identify those we may have missed)

Patient Barriers

☒ **Lack of knowledge:** Patient lacks the knowledge to understand and/or manage their condition
☐ **Disability:** Disability that makes getting health care difficult
☒ **Co-morbidity:** Medical or mental health problems that make getting care difficult
☒ **Not a priority:** Other issues take priority over care
☐ **Fear:** Fear about any aspect of health or health-related care
☐ **Attitudes toward providers:** Perceptions about health care providers that impact receiving care
☒ **Perceptions/Beliefs about test or treatment:** Personal or cultural beliefs that effect receiving care

External Barriers
☒ **Housing:** Worrying about housing during health care
☒ **Transportation:** Difficulty getting from home to health care site
☐ **Location of facility:** Distance from health care facility even if transportation is available
☒ **Social support:** Lacks a person/community for assistance during health care
☐ **Child care:** Not having child care when needed during health care
☐ **Adult care:** Difficulty finding support for other family members during health care
☐ **Employment demands:** Work demands make getting health care difficult
☐ **Insurance:** Paying for direct aspects of health care is a problem
☒ **Financial problems:** Dealing with financial problems (not directly related to health care) is interfering with receiving health care

System Barriers

☐ **Literacy:** Difficulty understanding written communication from the health care system
☒ **Language:** Not sharing a common language for communication
☒ **Problems with scheduling:** Difficulty scheduling and/or coordinating appointments
☒ **Communication with providers:** Lacks understanding of the information provided
☒ **System proactive:** Difficulty accessing appropriate providers or lack of follow-up for missed or cancelled appointments

4. Theories or frameworks used to inform the development of the intervention:

Chronic Care Model. Transtheroretical Model (Stages of Change), Self-efficacy, Theory of Planned Behavior, Social Cognitive Theory, Pender's Health Promotion Model

Percac-Lima et al. 2015. Patient Navigation Based on Predictive Modeling Decreases No-Show Rates in Cancer Care. *Cancer.* 121(10):1662-70.

Below please find a summary of the details we have extracted from your manuscript. Please confirm that we have reported accurate descriptions of the intervention aim and the actions taken by the case manager. In addition, please confirm whether we have accurately indicated all of the barriers targeted by the intervention, or whether additional barriers were also addressed. There are four (4) questions in total.

1. Intervention Aim: Improve outpatient compliance with appointments for patients with cancer who were identified by predictive modeling as being at high risk of missing a scheduled appointment.

Please denote here if the overall aim was different

2. Case Manager Duties: (Please check the boxes below to confirm the duties we extracted from the manuscript and to identify those we may have missed)

- ☐ Provide education (written or verbal)
- ☐ Attend patient appointments
- ☐ Improve patient communication
- ☐ Act as an interpreter
- ☐ Provide social/emotional support
- ☐ Link patients to social resources
- ☐ Link patients to financial resources
- ☐ Schedule visits
- ☐ Facilitate referrals
- ☒ Provide information to healthcare provider
- ☐ Support self-management/reinforce skills
- ☐ Help with patient goal setting
- ☐ Link patients to billing/insurance personnel
- ☐ Liaise with employer to make sure health needs are met
- ☐ Monitor attendance & follow-up after missed appointments
- ☒ Other: Reminders prior to appointment

Of the duties listed above, ***please identify*** those which you feel had the greatest impact or should be included in the development of future navigator interventions (list below or highlight above).

Please identify most important duties here

Percac-Lima et al. 2015. Patient Navigation Based on Predictive Modeling Decreases No-Show Rates in Cancer Care. ***Cancer.*** **121(10):1662-70.**

3. Barriers targeted by program: (Please check the boxes below to confirm that we have accurately identified the barriers targeted by the intervention and to identify those we may have missed)

Patient Barriers

☐ **Lack of knowledge:** Patient lacks the knowledge to understand and/or manage their condition
☐ **Disability:** Disability that makes getting health care difficult
☐ **Co-morbidity:** Medical or mental health problems that make getting care difficult
☐ **Not a priority:** Other issues take priority over care
☐ **Fear:** Fear about any aspect of health or health-related care
☐ **Attitudes toward providers:** Perceptions about health care providers that impact receiving care
☐ **Perceptions/Beliefs about test or treatment:** Personal or cultural beliefs that effect receiving care

External Barriers
☐ **Housing:** Worrying about housing during health care
☐ **Transportation:** Difficulty getting from home to health care site
☐ **Location of facility:** Distance from health care facility even if transportation is available
☐ **Social support:** Lacks a person/community for assistance during health care
☐ **Child care:** Not having child care when needed during health care
☐ **Adult care:** Difficulty finding support for other family members during health care
☐ **Employment demands:** Work demands make getting health care difficult
☐ **Insurance:** Paying for direct aspects of health care is a problem
☐ **Financial problems:** Dealing with financial problems (not directly related to health care) is interfering with receiving health care

System Barriers

☐ **Literacy:** Difficulty understanding written communication from the health care system
☒ **Language:** Not sharing a common language for communication
☐ **Problems with scheduling:** Difficulty scheduling and/or coordinating appointments
☐ **Communication with providers:** Lacks understanding of the information provided
☐ **System proactive:** Difficulty accessing appropriate providers or lack of follow-up for missed or cancelled appointments

4. Theories or frameworks used to inform the development of the intervention:

Please identify any important theories or frameworks here

Metsch et al. 2015. Effects of a Brief Case Management Intervention Linking People With HIV to Oral Health Care: Project SMILE. *Am J Public Health.* 105(1):77-84.

Below please find a summary of the details we have extracted from your manuscript. Please confirm that we have reported accurate descriptions of the intervention aim and the actions taken by the case manager. In addition, please confirm whether we have accurately indicated all of the barriers targeted by the intervention, or whether additional barriers were also addressed. There are four (4) questions in total.

1. Intervention Aim: Increase access to and use of oral health services among low-income individuals with HIV.

Please denote here if the overall aim was different<br><br>the efficacy of a case management intervention that linked individuals to dental care

2. Case Manager Duties: (Please check the boxes below to confirm the duties we extracted from the manuscript and to identify those we may have missed)

- ☒ Provide education (written or verbal)
- ☐ Attend patient appointments
- ☐ Improve patient communication
- ☐ Act as an interpreter
- ☐ Provide social/emotional support
- ☒ Link patients to social resources
- ☐ Link patients to financial resources
- ☐ Monitor attendance & follow-up after missed appointments
- ☒ Other: Arrange transportation
- ☒ Schedule visits
- ☒ Facilitate referrals
- ☐ Provide information to healthcare provider
- ☒ Support self-management/reinforce skills
- ☐ Help with patient goal setting
- ☒ Link patients to billing/insurance personnel
- ☐ Liaise with employer to make sure health needs are met

Of the duties listed above, please identify those which you feel had the greatest impact or should be included in the development of future navigator interventions (list below or highlight above).

Please identify most important duties here<br><br>Identifying individual and structural barriers to obtaining care<br>addressing structural barriers and/or linking participants to case management services

Metsch et al. 2015. Effects of a Brief Case Management Intervention Linking People With HIV to Oral Health Care: Project SMILE. *Am J Public Health.* 105(1):77-84.

3. Barriers targeted by program: (Please check the boxes below to confirm that we have accurately identified the barriers targeted by the intervention and to identify those we may have missed)

Patient Barriers

☒ **Lack of knowledge:** Patient lacks the knowledge to understand and/or manage their condition
☐ **Disability:** Disability that makes getting health care difficult
☐ **Co-morbidity:** Medical or mental health problems that make getting care difficult
☐ **Not a priority:** Other issues take priority over care
☐ **Fear:** Fear about any aspect of health or health-related care
☐ **Attitudes toward providers:** Perceptions about health care providers that impact receiving care
☐ **Perceptions/Beliefs about test or treatment:** Personal or cultural beliefs that effect receiving care

External Barriers

☐ **Housing:** Worrying about housing during health care
☒ **Transportation:** Difficulty getting from home to health care site
☐ **Location of facility:** Distance from health care facility even if transportation is available
☐ **Social support:** Lacks a person/community for assistance during health care
☐ **Child care:** Not having child care when needed during health care
☐ **Adult care:** Difficulty finding support for other family members during health care
☐ **Employment demands:** Work demands make getting health care difficult
☒ **Insurance:** Paying for direct aspects of health care is a problem
☐ **Financial problems:** Dealing with financial problems (not directly related to health care) is interfering with receiving health care

System Barriers

☐ **Literacy:** Difficulty understanding written communication from the health care system
☐ **Language:** Not sharing a common language for communication
☐ **Problems with scheduling:** Difficulty scheduling and/or coordinating appointments
☐ **Communication with providers:** Lacks understanding of the information provided
☒ **System proactive:** Difficulty accessing appropriate providers or lack of follow-up for missed or cancelled appointments

4. Theories or frameworks used to inform the development of the intervention:

Theories of Empowerment and Self-Efficacy (from Gardner 2005)

Please identify any important theories or frameworks here

Prezio et al. 2013. Community Diabetes Education (CoDE) for uninsured Mexican Americans: A randomized controlled trial of a culturally tailored diabetes education and management program led by a community health worker. ***Diabetes Res Clin Pract.*** **100:19-28.**

Below please find a summary of the details we have extracted from your manuscript. Please confirm that we have reported accurate descriptions of the intervention aim and the actions taken by the community health worker. In addition, please confirm whether we have accurately indicated all of the barriers targeted by the intervention, or whether additional barriers were also addressed. There are four (4) questions in total.

1. Intervention Aim: Provide access to Diabetes self-management education for uninsured patients.

Please denote here if the overall aim was different

2. Community Health Worker Duties: (Please check the boxes below to confirm the duties we extracted from the manuscript and to identify those we may have missed)

- ☒ Provide education (written or verbal)
- ☐ Attend patient appointments
- ☐ Improve patient communication
- ☐ Act as an interpreter
- ☐ Provide social/emotional support
- ☐ Link patients to social resources
- ☐ Link patients to financial resources
- ☒ Schedule visits
- ☒ Facilitate referrals
- ☐ Provide information to healthcare provider
- ☒ Support self-management/reinforce skills
- ☐ Help with patient goal setting
- ☐ Link patients to billing/insurance personnel
- ☐ Liaise with employer to make sure health needs are met
- ☐ Monitor attendance & follow-up after missed appointments
- ☐ Other:

Of the duties listed above, please identify those which you feel had the greatest impact or should be included in the development of future navigator interventions (list below or highlight above).

Please identify most important duties here

Prezio et al. 2013. Community Diabetes Education (CoDE) for uninsured Mexican Americans: A randomized controlled trial of a culturally tailored diabetes education and management program led by a community health worker. *Diabetes Res Clin Pract.* 100:19-28.

3. Barriers targeted by program: (Please check the boxes below to confirm that we have accurately identified the barriers targeted by the intervention and to identify those we may have missed)

Patient Barriers

☒ **Lack of knowledge:** Patient lacks the knowledge to understand and/or manage their condition
☐ **Disability:** Disability that makes getting health care difficult
☐ **Co-morbidity:** Medical or mental health problems that make getting care difficult
☐ **Not a priority:** Other issues take priority over care
☐ **Fear:** Fear about any aspect of health or health-related care
☐ **Attitudes toward providers:** Perceptions about health care providers that impact receiving care
☐ **Perceptions/Beliefs about test or treatment:** Personal or cultural beliefs that effect receiving care

External Barriers

☐ **Housing:** Worrying about housing during health care
☐ **Transportation:** Difficulty getting from home to health care site
☐ **Location of facility:** Distance from health care facility even if transportation is available
☐ **Social support:** Lacks a person/community for assistance during health care
☐ **Child care:** Not having child care when needed during health care
☐ **Adult care:** Difficulty finding support for other family members during health care
☐ **Employment demands:** Work demands make getting health care difficult
☒ **Insurance:** Paying for direct aspects of health care is a problem
☐ **Financial problems:** Dealing with financial problems (not directly related to health care) is interfering with receiving health care

System Barriers

☒ **Literacy:** Difficulty understanding written communication from the health care system
☒ **Language:** Not sharing a common language for communication
☒ **Problems with scheduling:** Difficulty scheduling and/or coordinating appointments
☐ **Communication with providers:** Lacks understanding of the information provided
☐ **System proactive:** Difficulty accessing appropriate providers or lack of follow-up for missed or cancelled appointments

4. Theories or frameworks used to inform the development of the intervention:

Social Cognitive Theory

Please identify any other important theories or frameworks here

Sullivan et al. 2012. Impact of Navigators on Completion of Steps in the Kidney Transplant Process: A Randomized, Controlled Trial. *Clin J Am Soc Nephrol.* 7: 1639–1645.

Below please find a summary of the details we have extracted from your manuscript. Please confirm that we have reported accurate descriptions of the intervention aim and the actions taken by the patient navigator. In addition, please confirm whether we have accurately indicated all of the barriers targeted by the intervention, or whether additional barriers were also addressed. There are four (4) questions in total.

1. Intervention Aim: Help patients complete transplant process steps in a more efficient and equitable manner.

Please denote here if the overall aim was different

2. Navigator Duties: (Please check the boxes below to confirm the duties we extracted from the manuscript and to identify those we may have missed)

- ☒ Provide education (written or verbal)
- ☐ Attend patient appointments
- ☒ Improve patient communication
- ☐ Act as an interpreter
- ☒ Provide social/emotional support
- ☐ Link patients to social resources
- ☐ Link patients to financial resources
- ☒ Monitor attendance & follow-up after missed appointments
- ☒ Other: Arrange transportation
- ☐ Schedule visits
- ☒ Facilitate referrals
- ☐ Provide information to healthcare provider
- ☐ Support self-management/reinforce skills
- ☐ Help with patient goal setting
- ☐ Link patients to billing/insurance personnel
- ☐ Liaise with employer to make sure health needs are met

Of the duties listed above, please identify those which you feel had the greatest impact or should be included in the development of future navigator interventions (list below or highlight above).

Please identify most important duties here

Sullivan et al. 2012. Impact of Navigators on Completion of Steps in the Kidney Transplant Process: A Randomized, Controlled Trial. *Clin J Am Soc Nephrol.* 7: 1639–1645.

3. Barriers targeted by program: (Please check the boxes below to confirm that we have accurately identified the barriers targeted by the intervention and to identify those we may have missed)

Patient Barriers

☒ **Lack of knowledge:** Patient lacks the knowledge to understand and/or manage their condition
☐ **Disability:** Disability that makes getting health care difficult
☐ **Co-morbidity:** Medical or mental health problems that make getting care difficult
☐ **Not a priority:** Other issues take priority over care
☐ **Fear:** Fear about any aspect of health or health-related care
☐ **Attitudes toward providers:** Perceptions about health care providers that impact receiving care
☐ **Perceptions/Beliefs about test or treatment:** Personal or cultural beliefs that effect receiving care

External Barriers

☐ **Housing:** Worrying about housing during health care
☒ **Transportation:** Difficulty getting from home to health care site
☐ **Location of facility:** Distance from health care facility even if transportation is available
☒ **Social support:** Lacks a person/community for assistance during health care
☐ **Child care:** Not having child care when needed during health care
☐ **Adult care:** Difficulty finding support for other family members during health care
☐ **Employment demands:** Work demands make getting health care difficult
☐ **Insurance:** Paying for direct aspects of health care is a problem
☐ **Financial problems:** Dealing with financial problems (not directly related to health care) is interfering with receiving health care

System Barriers

☐ **Literacy:** Difficulty understanding written communication from the health care system
☐ **Language:** Not sharing a common language for communication
☐ **Problems with scheduling:** Difficulty scheduling and/or coordinating appointments
☒ **Communication with providers:** Information provided is unclear or patient lacks understanding
☒ **System proactive:** Difficulty accessing appropriate providers or lack of follow-up for missed or cancelled appointments

4. Theories or frameworks used to inform the development of the intervention:

Please identify any important theories or frameworks here

Fiscella et al. 2012. Patient Navigation for Breast and Colorectal Cancer Treatment: A Randomized Trial. *Cancer Epidemiol Biomarkers Prev.* 21(10):1673–1681.

Below please find a summary of the details we have extracted from your manuscript. Please confirm that we have reported accurate descriptions of the intervention aim and the actions taken by the patient navigator. In addition, please confirm whether we have accurately indicated all of the barriers targeted by the intervention, or whether additional barriers were also addressed. There are four (4) questions in total.

1. Intervention Aim: Optimize treatment of patients diagnosed with cancer by providing a patient navigator to act as a guide and coach during cancer treatment.

Please denote here if the overall aim was different

2. Navigator Duties: (Please check the boxes below to confirm the duties we extracted from the manuscript and to identify those we may have missed)

- ☒ Provide education (written or verbal)
- ☒ Attend patient appointments
- ☒ Improve patient communication
- ☐ Act as an interpreter
- ☐ Provide social/emotional support
- ☒ Link patients to social resources
- ☒ Link patients to financial resources
- ☒ Schedule visits
- ☐ Facilitate referrals
- ☒ Provide information to healthcare provider
- ☐ Support self-management/reinforce skills
- ☐ Help with patient goal setting
- ☐ Link patients to billing/insurance personnel
- ☐ Liaise with employer to make sure health needs are met
- ☐ Monitor attendance & follow-up after missed appointments
- ☐ Other:

Of the duties listed above, please identify those which you feel had the greatest impact or should be included in the development of future navigator interventions (list below or highlight above).

Please identify most important duties here

Fiscella et al. 2012. Patient Navigation for Breast and Colorectal Cancer Treatment: A Randomized Trial. ***Cancer Epidemiol Biomarkers Prev.*** **21(10):1673–1681.**

3. Barriers targeted by program: (Please check the boxes below to confirm that we have accurately identified the barriers targeted by the intervention and to identify those we may have missed)

Patient Barriers

☒ **Lack of knowledge:** Patient lacks the knowledge to understand and/or manage their condition
☐ **Disability:** Disability that makes getting health care difficult
☐ **Co-morbidity:** Medical or mental health problems that make getting care difficult
☐ **Not a priority:** Other issues take priority over care
☐ **Fear:** Fear about any aspect of health or health-related care
☐ **Attitudes toward providers:** Perceptions about health care providers that impact receiving care
☐ **Perceptions/Beliefs about test or treatment:** Personal or cultural beliefs that effect receiving care

External Barriers
☐ **Housing:** Worrying about housing during health care
☐ **Transportation:** Difficulty getting from home to health care site
☐ **Location of facility:** Distance from health care facility even if transportation is available
☒ **Social support:** Lacks a person/community for assistance during health care
☐ **Child care:** Not having child care when needed during health care
☐ **Adult care:** Difficulty finding support for other family members during health care
☐ **Employment demands:** Work demands make getting health care difficult
☐ **Insurance:** Paying for direct aspects of health care is a problem
☒ **Financial problems:** Dealing with financial problems (not directly related to health care) is interfering with receiving health care

System Barriers

☐ **Literacy:** Difficulty understanding written communication from the health care system
☒ **Language:** Not sharing a common language for communication
☒ **Problems with scheduling:** Difficulty scheduling and/or coordinating appointments
☒ **Communication with providers:** Lacks understanding of the information provided
☒ **System proactive:** Difficulty accessing appropriate providers or lack of follow-up for missed or cancelled appointments

4. Theories or frameworks used to inform the development of the intervention:

Please identify any important theories or frameworks here

Kneipp et al. 2011. Public Health Nursing Case Management for Women Receiving Temporary Assistance for Needy Families: A Randomized Controlled Trial Using Community-Based Participatory Research. *Am J Public Health.* 101(9): 1759–1768.

Below please find a summary of the details we have extracted from your manuscript. Please confirm that we have reported accurate descriptions of the intervention aim and the actions taken by the public health nurse. In addition, please confirm whether we have accurately indicated all of the barriers targeted by the intervention, or whether additional barriers were also addressed. There are four (4) questions in total.

1. Intervention Aim: Increase rates of health care visits for mental health and chronic health conditions and increase the ability to navigate the Medicaid system.

Please denote here if the overall aim was different

2. Public Health Nurse Duties: (Please check the boxes below to confirm the duties we extracted from the manuscript and to identify those we may have missed)

- ☒ Provide education (written or verbal)
- ☐ Attend patient appointments
- ☐ Improve patient communication
- ☐ Act as an interpreter
- ☒ Provide social/emotional support
- ☒ Link patients to social resources
- ☐ Link patients to financial resources
- ☐ Monitor attendance & follow-up after missed appointments
- ☐ Other:
- ☒ Schedule visits
- ☒ Facilitate referrals
- ☐ Provide information to healthcare provider
- ☐ Support self-management/reinforce skills
- ☒ Help with patient goal setting
- ☐ Link patients to billing/insurance personnel
- ☒ Liaise with employer to make sure health needs are met

Of the duties listed above, please identify those which you feel had the greatest impact or should be included in the development of future navigator interventions (list below or highlight above).

Please identify most important duties here

Kneipp et al. 2011. Public Health Nursing Case Management for Women Receiving Temporary Assistance for Needy Families: A Randomized Controlled Trial Using Community-Based Participatory Research. *Am J Public Health.* 101(9): 1759–1768.

3. Barriers targeted by program: (Please check the boxes below to confirm that we have accurately identified the barriers targeted by the intervention and to identify those we may have missed)

Patient Barriers

☒ **Lack of knowledge:** Patient lacks the knowledge to understand and/or manage their condition
☐ **Disability:** Disability that makes getting health care difficult
☐ **Co-morbidity:** Medical or mental health problems that make getting care difficult
☐ **Not a priority:** Other issues take priority over care
☐ **Fear:** Fear about any aspect of health or health-related care
☐ **Attitudes toward providers:** Perceptions about health care providers that impact receiving care
☐ **Perceptions/Beliefs about test or treatment:** Personal or cultural beliefs that effect receiving care

External Barriers
☐ **Housing:** Worrying about housing during health care
☐ **Transportation:** Difficulty getting from home to health care site
☐ **Location of facility:** Distance from health care facility even if transportation is available
☒ **Social support:** Lacks a person/community for assistance during health care
☐ **Child care:** Not having child care when needed during health care
☐ **Adult care:** Difficulty finding support for other family members during health care
☒ **Employment demands:** Work demands make getting health care difficult
☒ **Insurance:** Paying for direct aspects of health care is a problem
☒ **Financial problems:** Dealing with financial problems (not directly related to health care) is interfering with receiving health care

System Barriers

☐ **Literacy:** Difficulty understanding written communication from the health care system
☐ **Language:** Not sharing a common language for communication
☒ **Problems with scheduling:** Difficulty scheduling and/or coordinating appointments
☐ **Communication with providers:** Information provided is unclear or patient lacks understanding
☐ **System proactive:** Difficulty accessing appropriate providers or lack of follow-up for missed or cancelled appointments

4. Theories or frameworks used to inform the development of the intervention:

Transactional Model of Stress and Coping

Please identify any other important theories or frameworks here

Spencer et al. 2011. Effectiveness of a Community Health Worker Intervention Among African American and Latino Adults With Type 2 Diabetes: A Randomized Controlled Trial. *Am J Public Health.* 101(12):2253-60.

Below please find a summary of the details we have extracted from your manuscript. Please confirm that we have reported accurate descriptions of the intervention aim and the actions taken by the community health worker. In addition, please confirm whether we have accurately indicated all of the barriers targeted by the intervention, or whether additional barriers were also addressed. There are four (4) questions in total.

1. Intervention Aim: Improve diabetes self-management behaviours.

Please denote here if the overall aim was different

2. Community Health Worker Duties: (Please check the boxes below to confirm the duties we extracted from the manuscript and to identify those we may have missed)

- ☐ Provide education (written or verbal)
- ☐ Attend patient appointments
- ☒ Improve patient communication
- ☐ Act as an interpreter
- ☒ Provide social/emotional support
- ☐ Link patients to social resources
- ☐ Link patients to financial resources
- ☐ Schedule visits
- ☒ Facilitate referrals
- ☐ Provide information to healthcare provider
- ☒ Support self-management/reinforce skills
- ☒ Help with patient goal setting
- ☐ Link patients to billing/insurance personnel
- ☐ Liaise with employer to make sure health needs are met
- ☐ Monitor attendance & follow-up after missed appointments
- ☐ Other:

Of the duties listed above, please identify those which you feel had the greatest impact or should be included in the development of future navigator interventions (list below or highlight above).

Please identify most important duties here

Spencer et al. 2011. Effectiveness of a Community Health Worker Intervention Among African American and Latino Adults With Type 2 Diabetes: A Randomized Controlled Trial. *Am J Public Health.* 101(12):2253-60.

3. Barriers targeted by program: (Please check the boxes below to confirm that we have accurately identified the barriers targeted by the intervention and to identify those we may have missed)

Patient Barriers

☐ **Lack of knowledge:** Patient lacks the knowledge to understand and/or manage their condition
☐ **Disability:** Disability that makes getting health care difficult
☐ **Co-morbidity:** Medical or mental health problems that make getting care difficult
☐ **Not a priority:** Other issues take priority over care
☐ **Fear:** Fear about any aspect of health or health-related care
☐ **Attitudes toward providers:** Perceptions about health care providers that impact receiving care
☐ **Perceptions/Beliefs about test or treatment:** Personal or cultural beliefs that effect receiving care

External Barriers
☐ **Housing:** Worrying about housing during health care
☐ **Transportation:** Difficulty getting from home to health care site
☐ **Location of facility:** Distance from health care facility even if transportation is available
☒ **Social support:** Lacks a person/community for assistance during health care
☐ **Child care:** Not having child care when needed during health care
☐ **Adult care:** Difficulty finding support for other family members during health care
☐ **Employment demands:** Work demands make getting health care difficult
☒ **Insurance:** Paying for direct aspects of health care is a problem
☐ **Financial problems:** Dealing with financial problems (not directly related to health care) is interfering with receiving health care

System Barriers

☐ **Literacy:** Difficulty understanding written communication from the health care system
☒ **Language:** Not sharing a common language for communication
☐ **Problems with scheduling:** Difficulty scheduling and/or coordinating appointments
☒ **Communication with providers:** Lacks understanding of the information provided
☒ **System proactive:** Difficulty accessing appropriate providers or lack of follow-up for missed or cancelled appointments

4. Theories or frameworks used to inform the development of the intervention:

Empowerment Theory

Please identify any other important theories or frameworks here

Ell et al. 2009. Cancer Treatment Adherence among Low-Income Women with Breast or Gynecologic Cancer: A Randomized Controlled Trial of Patient Navigation. 115(19): 4606–4615.

Below please find a summary of the details we have extracted from your manuscript. Please confirm that we have reported accurate descriptions of the intervention aim and the actions taken by the patient navigator. In addition, please confirm whether we have accurately indicated all of the barriers targeted by the intervention, or whether additional barriers were also addressed. There are four (4) questions in total.

1. Intervention Aim: Improve treatment and follow-up access and adherence by influencing predisposing (knowledge/attitudes), reinforcing (social support/cues to action), and enabling (barrier reduction skill).

Please denote here if the overall aim was different

2. Navigator Duties: (Please check boxes to confirm the duties we extracted from the manuscript and to identify those we may have missed)

- ☒ Provide education (written or verbal)
- ☐ Attend patient appointments
- ☐ Improve patient communication
- ☐ Act as an interpreter
- ☐ Provide social/emotional support
- ☐ Link patients to social resources
- ☐ Link patients to financial resources
- ☐ Schedule visits
- ☐ Facilitate referrals
- ☐ Provide information to healthcare provider
- ☒ Support self-management/reinforce skills
- ☐ Help with patient goal setting
- ☐ Link patients to billing/insurance personnel
- ☐ Liaise with employer to make sure health needs are met
- ☐ Monitor attendance & follow-up after missed appointments
- ☐ Other:

Of the duties listed above, please identify those which you feel had the greatest impact or should be included in the development of future navigator interventions (list below or highlight above).

Please identify most important duties here

Ell et al. 2009. Cancer Treatment Adherence among Low-Income Women with Breast or Gynecologic Cancer: A Randomized Controlled Trial of Patient Navigation. 115(19): 4606–4615.

3. Barriers targeted by program: (Please check boxes to confirm that we have accurately identified the barriers targeted by the intervention and to identify those we may have missed)

Patient Barriers

☒ **Lack of knowledge:** Patient lacks the knowledge to understand and/or manage their condition
☐ **Disability:** Disability that makes getting health care difficult
☐ **Co-morbidity:** Medical or mental health problems that make getting care difficult
☐ **Not a priority:** Other issues take priority over care
☐ **Fear:** Fear about any aspect of health or health-related care
☐ **Attitudes toward providers:** Perceptions about health care providers that impact receiving care
☐ **Perceptions/Beliefs about test or treatment:** Personal or cultural beliefs that effect receiving care

External Barriers
☐ **Housing:** Worrying about housing during health care
☐ **Transportation:** Difficulty getting from home to health care site
☐ **Location of facility:** Distance from health care facility even if transportation is available
☒ **Social support:** Lacks a person/community for assistance during health care
☐ **Child care:** Not having child care when needed during health care
☐ **Adult care:** Difficulty finding support for other family members during health care
☐ **Employment demands:** Work demands make getting health care difficult
☐ **Insurance:** Paying for direct aspects of health care is a problem
☐ **Financial problems:** Dealing with financial problems (not directly related to health care) is interfering with receiving health care

System Barriers

☐ **Literacy:** Difficulty understanding written communication from the health care system
☒ **Language:** Not sharing a common language for communication
☐ **Problems with scheduling:** Difficulty scheduling and/or coordinating appointments
☐ **Communication with providers:** Lacks understanding of the information provided
☐ **System proactive:** Difficulty accessing appropriate providers or lack of follow-up for missed or cancelled appointments

4. Theories or frameworks used to inform the development of the intervention:

Health Belief Model
Socio-Cultural Explanatory Theory

Please identify any other important theories or frameworks here

Wohl et al. 2006. A Randomized Trial of Directly Administered Antiretroviral Therapy and Adherence Case Management Intervention. *Clin Infect Dis.* 42:1619–27.

Below please find a summary of the details we have extracted from your manuscript. Please confirm that we have reported accurate descriptions of the intervention aim and the actions taken by the case manager. In addition, please confirm whether we have accurately indicated all of the barriers targeted by the intervention, or whether additional barriers were also addressed. There are four (4) questions in total.

1. Intervention Aim: Overcome barriers to treatment adherence while engaging in case management activities.

Please denote here if the overall aim was different

To use a brief case management intervention to imporve adherence to antiretroviral therapy among persons living with HIV in Los Angeles County.

2. Case Manager Duties: (Please check the boxes below to confirm the duties we extracted from the manuscript and to identify those we may have missed)

- ☒ Provide education (written or verbal)
- ☐ Attend patient appointments
- ☒ Improve patient communication
- ☐ Act as an interpreter
- ☒ Provide social/emotional support
- ☒ Link patients to social resources
- ☒ Link patients to financial resources
- ☐ Schedule visits
- ☒ Facilitate referrals
- ☒ Provide information to healthcare provider
- ☒ Support self-management/reinforce skills
- ☒ Help with patient goal setting
- ☒ Link patients to billing/insurance personnel
- ☐ Liaise with employer to make sure health needs are met
- ☐ Monitor attendance & follow-up after missed appointments
- ☐ Other:

Of the duties listed above, please identify those which you feel had the greatest impact or should be included in the development of future navigator interventions (list below or highlight above).

Please identify most important duties here

Wohl et al. 2006. A Randomized Trial of Directly Administered Antiretroviral Therapy and Adherence Case Management Intervention. *Clin Infect Dis.* 42:1619–27.

3. Barriers targeted by program: (Please check the boxes below to confirm that we have accurately identified the barriers targeted by the intervention and to identify those we may have missed)

Patient Barriers

☒ **Lack of knowledge:** Patient lacks the knowledge to understand and/or manage their condition
☐ **Disability:** Disability that makes getting health care difficult
☒ **Co-morbidity:** Medical or mental health problems that make getting care difficult
☐ **Not a priority:** Other issues take priority over care
☐ **Fear:** Fear about any aspect of health or health-related care
☐ **Attitudes toward providers:** Perceptions about health care providers that impact receiving care
☐ **Perceptions/Beliefs about test or treatment:** Personal or cultural beliefs that effect receiving care

External Barriers
☒ **Housing:** Worrying about housing during health care
☒ **Transportation:** Difficulty getting from home to health care site
☐ **Location of facility:** Distance from health care facility even if transportation is available
☒ **Social support:** Lacks a person/community for assistance during health care
☒ **Child care:** Not having child care when needed during health care
☐ **Adult care:** Difficulty finding support for other family members during health care
☒ **Employment demands:** Work demands make getting health care difficult
☒ **Insurance:** Paying for direct aspects of health care is a problem
☒ **Financial problems:** Dealing with financial problems (not directly related to health care) is interfering with receiving health care

System Barriers

☒ **Literacy:** Difficulty understanding written communication from the health care system
☒ **Language:** Not sharing a common language for communication
☐ **Problems with scheduling:** Difficulty scheduling and/or coordinating appointments
☒ **Communication with providers:** Lacks understanding of the information provided
☐ **System proactive:** Difficulty accessing appropriate providers or lack of follow-up for missed or cancelled appointments

4. Theories or frameworks used to inform the development of the intervention:

Transtheoretical Model of Behaviour Change

Please identify any important theories or frameworks here

Gardener et al. 2005. Efficacy of a brief case management intervention to link recently diagnosed HIV-infected persons to care. *AIDS.* 19:423–431.

Below please find a summary of the details we have extracted from your manuscript. Please confirm that we have reported accurate descriptions of the intervention aim and the actions taken by the case manager. In addition, please confirm whether we have accurately indicated all of the barriers targeted by the intervention, or whether additional barriers were also addressed. There are four (4) questions in total.

1. Intervention Aim: Link HIV infected persons to HIV care, and to sustain this linkage for more than a single visit.

Please denote here if the overall aim was different

2. Case Manager Duties: (Please check the boxes below to confirm the duties we extracted from the manuscript and to identify those we may have missed)

- ☐ Provide education (written or verbal)
- ☐ Attend patient appointments
- ☐ Improve patient communication
- ☐ Act as an interpreter
- ☒ Provide social/emotional support
- ☐ Link patients to social resources
- ☐ Link patients to financial resources
- ☐ Schedule visits
- ☒ Facilitate referrals
- ☐ Provide information to healthcare provider
- ☒ Support self-management/reinforce skills
- ☐ Help with patient goal setting
- ☐ Link patients to billing/insurance personnel
- ☐ Liaise with employer to make sure health needs are met
- ☐ Monitor attendance & follow-up after missed appointments
- ☐ Other:

Of the duties listed above, please identify those which you feel had the greatest impact or should be included in the development of future navigator interventions (list below or highlight above).

Please identify most important duties here

Gardener et al. 2005. Efficacy of a brief case management intervention to link recently diagnosed HIV-infected persons to care. *AIDS.* 19:423–431.

3. Barriers targeted by program: (Please check the boxes below to confirm that we have accurately identified the barriers targeted by the intervention and to identify those we may have missed)

Patient Barriers

☐ **Lack of knowledge:** Patient lacks the knowledge to understand and/or manage their condition
☐ **Disability:** Disability that makes getting health care difficult
☐ **Co-morbidity:** Medical or mental health problems that make getting care difficult
☐ **Not a priority:** Other issues take priority over care
☐ **Fear:** Fear about any aspect of health or health-related care
☐ **Attitudes toward providers:** Perceptions about health care providers that impact receiving care
☐ **Perceptions/Beliefs about test or treatment:** Personal or cultural beliefs that effect receiving care

External Barriers

☐ **Housing:** Worrying about housing during health care
☐ **Transportation:** Difficulty getting from home to health care site
☐ **Location of facility:** Distance from health care facility even if transportation is available
☒ **Social support:** Lacks a person/community for assistance during health care
☐ **Child care:** Not having child care when needed during health care
☐ **Adult care:** Difficulty finding support for other family members during health care
☐ **Employment demands:** Work demands make getting health care difficult
☐ **Insurance:** Paying for direct aspects of health care is a problem
☐ **Financial problems:** Dealing with financial problems (not directly related to health care) is interfering with receiving health care

System Barriers

☐ **Literacy:** Difficulty understanding written communication from the health care system
☐ **Language:** Not sharing a common language for communication
☐ **Problems with scheduling:** Difficulty scheduling and/or coordinating appointments
☐ **Communication with providers:** Lacks understanding of the information provided
☒ **System proactive:** Difficulty accessing appropriate providers or lack of follow-up for missed or cancelled appointments

4. Theories or frameworks used to inform the development of the intervention:

Theories of Empowerment and Self-Efficacy

Please identify any other important theories or frameworks here

Gary et al. 2003. Randomized controlled trial of the effects of nurse case manager and community health worker interventions on risk factors for diabetes related complications in urban African Americans. *Preventive Medicine.* 37:23–32.

Below please find a summary of the details we have extracted from your manuscript. Please confirm that we have reported accurate descriptions of the intervention aim and the actions taken by the community health worker. In addition, please confirm whether we have accurately indicated all of the barriers targeted by the intervention, or whether additional barriers were also addressed. There are four (4) questions in total.

1. Intervention Aim: Improve diabetic control.

Please denote here if the overall aim was different

2. Community Health Worker Duties: (Please check the boxes below to confirm the duties we extracted from the manuscript and to identify those we may have missed)

- [x] Provide education (written or verbal)
- [ ] Attend patient appointments
- [ ] Improve patient communication
- [ ] Act as an interpreter
- [x] Provide social/emotional support
- [ ] Link patients to social resources
- [ ] Link patients to financial resources
- [x] Schedule visits
- [ ] Facilitate referrals
- [x] Provide information to healthcare provider
- [x] Support self-management/reinforce skills
- [ ] Help with patient goal setting
- [ ] Link patients to billing/insurance personnel
- [ ] Liaise with employer to make sure health needs are met
- [ ] Monitor attendance & follow-up after missed appointments
- [ ] Other:

Of the duties listed above, please identify those which you feel had the greatest impact or should be included in the development of future navigator interventions (list below or highlight above).

Please identify most important duties here

Gary et al. 2003. Randomized controlled trial of the effects of nurse case manager and community health worker interventions on risk factors for diabetes related complications in urban African Americans. ***Preventive Medicine.*** **37:23–32.**

3. Barriers targeted by program: (Please check the boxes below to confirm that we have accurately identified the barriers targeted by the intervention and to identify those we may have missed)

Patient Barriers

☒ **Lack of knowledge:** Patient lacks the knowledge to understand and/or manage their condition
☐ **Disability:** Disability that makes getting health care difficult
☐ **Co-morbidity:** Medical or mental health problems that make getting care difficult
☐ **Not a priority:** Other issues take priority over care
☐ **Fear:** Fear about any aspect of health or health-related care
☐ **Attitudes toward providers:** Perceptions about health care providers that impact receiving care
☐ **Perceptions/Beliefs about test or treatment:** Personal or cultural beliefs that effect receiving care

External Barriers

☐ **Housing:** Worrying about housing during health care
☐ **Transportation:** Difficulty getting from home to health care site
☐ **Location of facility:** Distance from health care facility even if transportation is available
☒ **Social support:** Lacks a person/community for assistance during health care
☐ **Child care:** Not having child care when needed during health care
☐ **Adult care:** Difficulty finding support for other family members during health care
☐ **Employment demands:** Work demands make getting health care difficult
☐ **Insurance:** Paying for direct aspects of health care is a problem
☐ **Financial problems:** Dealing with financial problems (not directly related to health care) is interfering with receiving health care

System Barriers

☐ **Literacy:** Difficulty understanding written communication from the health care system
☐ **Language:** Not sharing a common language for communication
☒ **Problems with scheduling:** Difficulty scheduling and/or coordinating appointments
☒ **Communication with providers:** Lacks understanding of the information provided
☐ **System proactive:** Difficulty accessing appropriate providers or lack of follow-up for missed or cancelled appointments

4. Theories or frameworks used to inform the development of the intervention:

Precede-Proceed

Please identify any other important theories or frameworks here

Svoren et al. 2003. Reducing Acute Adverse Outcomes for Youth with Type 1 Diabetes: A Randomized, Controlled Trial. *Pediatrics.* 12(4): 914-922.

Katz ML et al. Family-based psychoeducation and care ambassador intervention to improve glycemic control in youth with type 1 diabetes: a randomized trial Pediatric Diabetes 2014: 15: 142-150

Below please find a summary of the details we have extracted from your manuscript. Please confirm that we have reported accurate descriptions of the intervention aim and the actions taken by the Care Ambassador. In addition, please confirm whether we have accurately indicated all of the barriers targeted by the intervention, or whether additional barriers were also addressed. There are four (4) questions in total.

1. Intervention Aim: Help patients and their families receive ambulatory diabetes care as prescribed by the patient's usual diabetes health care team.

Please denote here if the overall aim was different

2. Care Ambassador Duties: (Please check the boxes below to confirm the duties we extracted from the manuscript and to identify those we may have missed)

- ☒ Provide education (written or verbal)
- ☐ Attend patient appointments
- ☐ Improve patient communication
- ☐ Act as an interpreter
- ☐ Provide social/emotional support
- ☐ Link patients to social resources
- ☐ Link patients to financial resources
- ☒ Schedule visits
- ☐ Facilitate referrals
- ☐ Provide information to healthcare provider
- ☐ Support self-management/reinforce skills
- ☐ Help with patient goal setting
- ☒ Link patients to billing/insurance personnel
- ☐ Liaise with employer to make sure health needs are met
- ☒ Monitor attendance & follow-up after missed appointments
- ☐ Other:

Of the duties listed above, please identify those which you feel had the greatest impact or should be included in the development of future navigator interventions (list below or highlight above).

Please identify most important duties here

Svoren et al. 2003. Reducing Acute Adverse Outcomes for Youth with Type 1 Diabetes: A Randomized, Controlled Trial. *Pediatrics.* 12(4): 914-922.

3. Barriers targeted by program: (Please check the boxes below to confirm that we have accurately identified the barriers targeted by the intervention and to identify those we may have missed)

Patient Barriers

☒ **Lack of knowledge:** Patient lacks the knowledge to understand and/or manage their condition
☐ **Disability:** Disability that makes getting health care difficult
☐ **Co-morbidity:** Medical or mental health problems that make getting care difficult
☐ **Not a priority:** Other issues take priority over care
☐ **Fear:** Fear about any aspect of health or health-related care
☐ **Attitudes toward providers:** Perceptions about health care providers that impact receiving care
☐ **Perceptions/Beliefs about test or treatment:** Personal or cultural beliefs that effect receiving care

External Barriers
☐ **Housing:** Worrying about housing during health care
☐ **Transportation:** Difficulty getting from home to health care site
☐ **Location of facility:** Distance from health care facility even if transportation is available
☐ **Social support:** Lacks a person/community for assistance during health care
☐ **Child care:** Not having child care when needed during health care
☐ **Adult care:** Difficulty finding support for other family members during health care
☐ **Employment demands:** Work demands make getting health care difficult
☒ **Insurance:** Paying for direct aspects of health care is a problem
☐ **Financial problems:** Dealing with financial problems (not directly related to health care) is interfering with receiving health care

System Barriers

☐ **Literacy:** Difficulty understanding written communication from the health care system
☐ **Language:** Not sharing a common language for communication
☒ **Problems with scheduling:** Difficulty scheduling and/or coordinating appointments
☐ **Communication with providers:** Lacks understanding of the information provided
☒ **System proactive:** Difficulty accessing appropriate providers or lack of follow-up for missed or cancelled appointments

4. Theories or frameworks used to inform the development of the intervention:

Please identify any important theories or frameworks here

Laffel et al. 1998. Changing the Process of Diabetes Care Improves Metabolic Outcomes and Reduces Hospitalizations. *Qual Manag Health Care.* 6(4): 53-62.

Below please find a summary of the details we have extracted from your manuscript. Please confirm that we have reported accurate descriptions of the intervention aim and the actions taken by the Care Ambassador. In addition, please confirm whether we have accurately indicated all of the barriers targeted by the intervention, or whether additional barriers were also addressed. There are four (4) questions in total.

1. Intervention Aim: Help patients and their families receive ambulatory diabetes care as prescribed by the patient's usual diabetes health care team.

Please denote here if the overall aim was different

2. Care Ambassador Duties: (Please check the boxes below to confirm the duties we extracted from the manuscript and to identify those we may have missed)

- ☐ Provide education (written or verbal)
- ☐ Attend patient appointments
- ☐ Improve patient communication
- ☐ Act as an interpreter
- ☐ Provide social/emotional support
- ☐ Link patients to social resources
- ☐ Link patients to financial resources
- ☒ Schedule visits
- ☐ Facilitate referrals
- ☐ Provide information to healthcare provider
- ☐ Support self-management/reinforce skills
- ☐ Help with patient goal setting
- ☒ Link patients to billing/insurance personnel
- ☐ Liaise with employer to make sure health needs are met
- ☒ Monitor attendance & follow-up after missed appointments
- ☐ Other:

Of the duties listed above, please identify those which you feel had the greatest impact or should be included in the development of future navigator interventions (list below or highlight above).

Please identify most important duties here

Laffel et al. 1998. Changing the Process of Diabetes Care Improves Metabolic Outcomes and Reduces Hospitalizations. *Qual Manag Health Care.* 6(4): 53-62.

3. Barriers targeted by program: (Please check the boxes below to confirm that we have accurately identified the barriers targeted by the intervention and to identify those we may have missed)

Patient Barriers

☐ **Lack of knowledge:** Patient lacks the knowledge to understand and/or manage their condition
☐ **Disability:** Disability that makes getting health care difficult
☐ **Co-morbidity:** Medical or mental health problems that make getting care difficult
☐ **Not a priority:** Other issues take priority over care
☐ **Fear:** Fear about any aspect of health or health-related care
☐ **Attitudes toward providers:** Perceptions about health care providers that impact receiving care
☐ **Perceptions/Beliefs about test or treatment:** Personal or cultural beliefs that effect receiving care

External Barriers

☐ **Housing:** Worrying about housing during health care
☐ **Transportation:** Difficulty getting from home to health care site
☐ **Location of facility:** Distance from health care facility even if transportation is available
☐ **Social support:** Lacks a person/community for assistance during health care
☐ **Child care:** Not having child care when needed during health care
☐ **Adult care:** Difficulty finding support for other family members during health care
☐ **Employment demands:** Work demands make getting health care difficult
☒ **Insurance:** Paying for direct aspects of health care is a problem
☐ **Financial problems:** Dealing with financial problems (not directly related to health care) is interfering with receiving health care

System Barriers

☐ **Literacy:** Difficulty understanding written communication from the health care system
☐ **Language:** Not sharing a common language for communication
☒ **Problems with scheduling:** Difficulty scheduling and/or coordinating appointments
☐ **Communication with providers:** Lacks understanding of the information provided
☒ **System proactive:** Difficulty accessing appropriate providers or lack of follow-up for missed or cancelled appointments

4. Theories or frameworks used to inform the development of the intervention:

Please identify any important theories or frameworks here
